# NEWS

**DOI:** 10.7189/jogh.08020201

**Published:** 2018-12

The *Journal of Global Health* has completed its first eight years of publishing editorials, viewpoints and research articles related to global health issues. From the very first issue, we also introduced *News* section. This section served as reader's digest of the developments that were relevant to world's seven major geographic regions, seven international agencies and seven key resources required to sustain the humanity. The *News* section summarized our choice of the most interesting events in the previous six months that could be classified under those headings. It intended to inform professionals active in the field of global health on the developments in this field.

In recent issues, we began to add further materials to our *News* section. These materials ranged from interesting meeting reports focused on global health, positive global initiatives, acknowledgements of professional success and awards received by our editors or Editorial Board members, to reflections on the activities of our Edinburgh University Global Health Society (EUGHS). These developments prompted us to rethink the role and usefulness of our *News* section. We realised that ubiquitous nature of news that can be found online today and the massive volume of the material makes it increasingly difficult for us to select or highlight the most relevant stories. Moreover, doing it twice a year means that many of the news we publish may already be out of date by the time we publish them, or superseded with new developments. We came to conclusion that the usefulness of the news digest in global health twice a year is of increasingly limited value.

**Figure:**
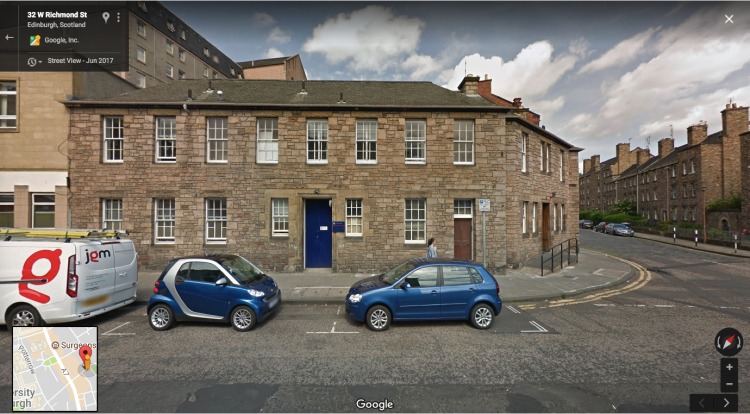
**Photo:** New location for the Centre for Global Health Research and World Health Organization's Collaborating Centre for Population Health Research and Training (Google Maps, June 2017)

**Figure:**
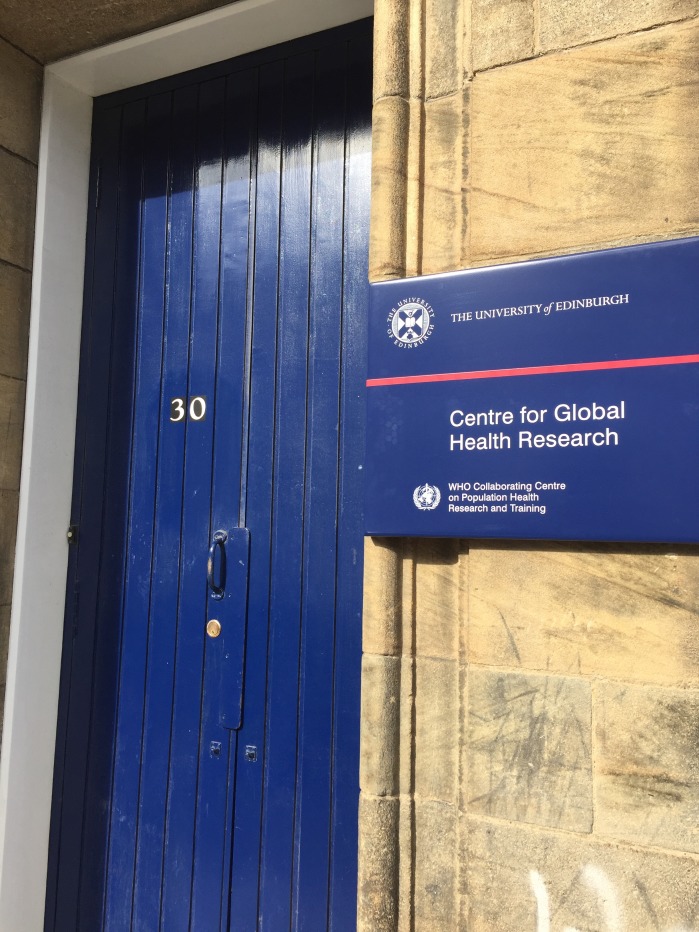
**Photo:** Entrance to the Centre for Global Health Research (photo courtesy of EUGHS)

On the other hand, there is a potentially very useful role for our *News* section in promoting the events, meetings and the work of global health centres in low- and middle-income countries and in high income countries alike. Therefore, from our first issue in 2019 we would like to invite our colleagues and readers from all world's regions to send us reports on the work of their research centres, departments or institutes, along with photographic material that can best support these reports. We think that this will become more useful and popular use of the space that we dedicated to reporting the news in the *Journal of Global Health.* From our next issue, we will start by reporting what is new in Edinburgh, where our Centre for Global Health Research and World Health Organization's Collaborating Centre for Population Health Research and Training has grown in size quite considerably over the past three years and moved to its own building at 30 West Richmond Street in Edinburgh, UK. We also invite and encourage news on the growth and development in capacity for global health research from other centres, especially those in poor settings, which are in position to make the most actual difference to health and well-being of their populations.

